# Injection of ligand-free gold and silver nanoparticles into murine embryos does not impact pre-implantation development

**DOI:** 10.3762/bjnano.5.80

**Published:** 2014-05-21

**Authors:** Ulrike Taylor, Wiebke Garrels, Annette Barchanski, Svea Peterson, Laszlo Sajti, Andrea Lucas-Hahn, Lisa Gamrad, Ulrich Baulain, Sabine Klein, Wilfried A Kues, Stephan Barcikowski, Detlef Rath

**Affiliations:** 1 Institute of Farm Animal Genetics, Friedrich-Loeffler-Institut, Federal Research Institute of Animal Health, Hoeltystrasse 10, 31535 Neustadt/Mariensee, Germany; 2 Nanotechnology Department, Laser Zentrum Hannover e.V., Hollerithallee 8, 30419 Hannover, Germany; 3 Institute for Biomedical Engineering, University of Rostock, Friedrich-Barnewitz-Strasse 4, 18119 Rostock, Germany; 4 Technical Chemistry I and Center for Nanointegration Duisburg-Essen (CENIDE), University of Duisburg-Essen, Universitätsstrasse 7, 45141 Essen, Germany

**Keywords:** biomedical application, confocal microscopy, gene expression, protein corona, toxicity

## Abstract

Intended exposure to gold and silver nanoparticles has increased exponentially over the last decade and will continue to rise due to their use in biomedical applications. In particular, reprotoxicological aspects of these particles still need to be addressed so that the potential impacts of this development on human health can be reliably estimated. Therefore, in this study the toxicity of gold and silver nanoparticles on mammalian preimplantation development was assessed by injecting nanoparticles into one blastomere of murine 2 cell-embryos, while the sister blastomere served as an internal control. After treatment, embryos were cultured and embryo development up to the blastocyst stage was assessed. Development rates did not differ between microinjected and control groups (gold nanoparticles: 67.3%, silver nanoparticles: 61.5%, sham: 66.2%, handling control: 79.4%). Real-time PCR analysis of six developmentally important genes (*BAX, BCL2L2, TP53, OCT4, NANOG, DNMT3A*) did not reveal an influence on gene expression in blastocysts. Contrary to silver nanoparticles, exposure to comparable Ag^+^-ion concentrations resulted in an immediate arrest of embryo development. In conclusion, the results do not indicate any detrimental effect of colloidal gold or silver nanoparticles on the development of murine embryos.

## Introduction

Gold and particularly silver are among the most commonly used materials for nanoparticle applications. They can be found in an increasing amount of consumer products [1], but they also emerge as materials for medical and biotechnological purposes [2–3]. Therefore, exposure to such particles – whether intentionally or unintentionally – is probable, and there is a need to profoundly understand any tentative side effects of such an exposure. In particular, this applies to sensitive areas like embryonic development where possibly occurring defects can be carried on to following generations.

While a range of rodent based studies describing in vivo nanoparticle distribution has been performed, only a few focused on placental crossing and their results are not entirely without ambiguity. In three studies, all of which utilize gold as material, nanoparticles were found to be stopped by the placental barrier [4–6]. The majority of authors, however, observed placental crossing. This encompasses studies of nanoparticles composed of gold [7–8], titanium dioxide [9–10], CdTe/CdS quantum dots [11], and polystyrene [12]. Thus, transplacental crossing of nanoparticles seems a likely scenario, but apparently depends on a variety of factors which are not well understood.

Embryo toxicology of nanoparticles has mainly been investigated on piscine embryos, mostly zebra fish. The tested materials include gold (AuNP) [13–15], silver (AgNP) [13,16–20], nickel (NiNP) [21], zinc oxide (ZnONP) [22–23], titanium dioxide (TiO_2_NP) [23–25], aluminium trioxide (Al_2_O_3_NP) [23] and copper (CuNP) [22,25]. Toxic effects were observed after exposure to AgNP, CuNP, ZnONP and NiNP. AuNP, TiO_2_NP and Al_2_O_3_NP, on the other hand, seemed to be inert, the sole exception being one study reporting an embryotoxic effect of gold clusters (nanoparticles < 2 nm) after applying an extraordinary high number dose of 10^14^ NP/embryo [15]. Comparatively well studied is also the effect of nanoparticles on avian embryos. Chicken embryos were exposed to nanoparticles made from gold [26], silver [27–30], silver–palladium alloy [31], and silver–copper alloy [30] by in ovo injection. Interestingly, no abnormal development was observed, except a low-grade inflammation of the embryonic liver after exposure to AgCu alloy nanoparticles. In mammals, almost all studies regarding embryotoxicity of nanoparticles were performed in mouse pups after exposing their mothers to titanium dioxide. While no information was given concerning the impact on early embryo development, it seems noteworthy that, regardless of the exposure route, in several studies pups of TiO_2_NP-treated mice showed abnormalities in the development of the nervous system [9,32–35]. Additionally, an increased risk of the pups to develop respiratory disease was noted [36]. Only one study so far investigated the effect of AgNP on the development of murine blastocysts in an in vitro culture model, noting increased apoptosis, decreased cell numbers and decreased implantation rates [37]. So far, AuNP have only been investigated once in a murine in vitro embryo culture model for their influence on blastocyst development [38]. In this study on chitosan nanoparticles morulae were co-incubated with AuNP as reference particles. No effect on embryo development was observed for the AuNP control. The listed studies show that embryo-toxicology of nanoparticles seems to depend on a variety of factors. One major point is the chemical composition of the particles. However, even particles in general viewed as rather noxious, like silver nanoparticles, did not always display the expected toxicity. Secondly, the outcome is apparently also dependent on the species tested. Nevertheless, due to considerable variations concerning the experimental set up and very different methods with regard to nanoparticle production – in particular the use of surfactants or stabilizers which may adsorb to the nanoparticle surface – a comparison is difficult and resilient conclusions remain elusive. In this context, it has been recently reported that the ligand type and its anchoring on the nanoparticle surface (physisorption or chemisorption) strongly affects the toxicity of AuNP on human embryonic kidney cells [39]. Another factor which has been shown to influence embryotoxicity is the size of the nanoparticles. For both AuNP and AgNP an increase in toxicity has been shown in conjuction with a decrease in size [13,40–41]. However, while AgNP remained embryotoxic even up to a size of 100 nm albeit in higher concentrations [13,40], AuNP only appear to be toxic if they are <2 nm [15,41], a size range at which gold nanoparticles consist of atom clusters.

**Figure 1:**
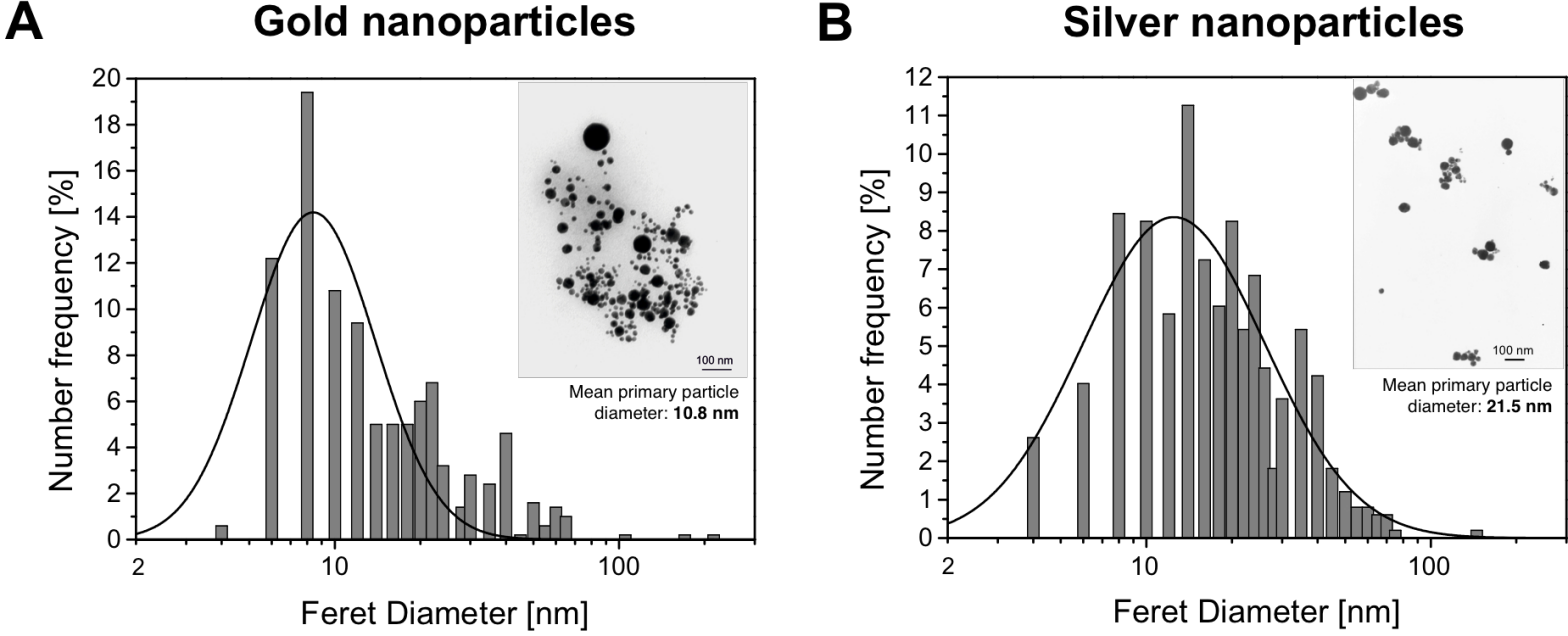
Number distribution of nanoparticle size including corresponding TEM micrographs as inserts of (A) gold nanoparticles and (B) silver nanoparticles.

In this work, AuNP and AgNP were chosen for testing. AuNP are currently viewed as promising agents for in vivo imaging purposes and might therefore be in frequent use in the near future. AgNP are already abundantly employed in applications for their antimicrobial properties. Both particle types also represent good models for exploring the extent to which toxic properties might change if the bulk material is converted to nanoscale. AuNP and AgNP can be viewed as being positioned on the opposite end of the spectrum concerning the ion release rate and the hazardous potential of the related metal ions, with gold assumed to be relatively bio-inert compared to the cytotoxicity of silver ions [42]. In order to exclude any cross-effects of stabilizers or reducing agents, which are difficult to exclude in precursor-based chemically produced gold and silver nanoparticles, the particles for this study were synthesized by laser ablation of a bulk solid target in water, which generates colloidal particles of maximal purity [43–45]. Without compromising this purity, monomodal and monodisperse gold colloids can easily be fabricated in micromolar saline water with defined AuNP sizes [46]. Alternatively, this method also provides particles displaying a relatively broad size range (Figure 1), which allows to screen for size related effects by offering several sizes at once to the exposed organism.

The produced nanoparticles were microinjected into one blastomere of a 2-cell murine embryo, while the sister blastomere remained untreated. This experimental set-up has formerly been shown to successfully work for the delivery of genetic material [47] and nanoparticles [48] into embryos. Alternatively, injection could have been performed on pronuclear stages. Such injections have been reported to provide a higher level of sensitivity for toxicological tests on embryos [49]. However, the injection into 2-cell-stage embryos allows for an internal control. This provides the opportunity to also detect sublethal effects, for instance, the interference with cell division mechanisms, a toxic effect already documented for gold nanoparticles [50].

There are other approaches of internalizing nanoparticles into a cellular environment, for example the spontaneous uptake during co-incubation or electroporation. Each of these have been proven to be successful [51–52], where the former has also been demonstrated to be applicable for particle uptake into an embryo [14]. However, spontaneous uptake can result into a very heterogeneous nanoparticle load among cells [51] while electroporation of embryos for nanoparticle internalization has not been attempted yet. Therefore, even if the injection of nanoparticles does not actually mirror nanoparticle exposure to preimplantation embryos in an in vivo setting, this method was chosen because it ensured the delivery of exact and therefore comparable amounts of nanoparticles.

Reverse amplification PCR (RT-PCR) of candidate messenger transcripts was selected as a method to highlight possible aberrations in development. The combination of the apoptosis regulator genes *BAX, BCL2L2* and *TP53* detects even subtle changes regarding apoptotic activity. *NANOG* and *OCT4* are exclusively expressed in cells of the inner cell mass (ICM). Thus, a decrease in mitosis or an interference with the potential to differentiate into ICM and trophoblast by cells derived from the injected blastomere will be picked up by a decrease in *NANOG* and *OCT4* expression. *DNMT3A* performs de-novo DNA methylation during the reprogramming phase of parental genomes. Therefore, changes in expression would point out interference with epigenetic processes. The administered dose of nanoparticles was calculated on the basis that an embryo would take up approximately 0.0004% of the particles [7] applied to the mother in clinically relevant settings [53–54]. The obtained results will aid to further clarify our conception of nanoparticle biocompatibility and differentiation of bio-response related to the particles.

## Results

### Characterization of gold and silver nanoparticles

Due to surface plasmon resonance (SPR), the fabricated gold nanoparticles exhibited a distinct absorption peak around 525 nm wavelength, while a peak at 400 nm was observed for silver nanoparticles. The mean primary particle diameter was determined by lognormal fitting of a particle number weighted diameter histogram derived from image processing of transmission electron microscopy (TEM) pictures and resulted in 11 nm for AuNP and 21 nm for AgNP. Number frequency distribution of the nanoparticle diameters as measured in the TEM are depicted in Figure 1A and Figure 1B. Featuring a surface charge AuNP are electrostatically stabilized in solution with a zeta potential of −25 mV, whereas the zeta potential of AgNP was determined to be −29 mV. All values including additional characteristics, and standard deviations are listed in Table 1.

**Table 1:** Gold and silver nanoparticle characteristics after synthesis by laser ablation.

	AuNP	AgNP

Absorption peak wavelength [nm]	525	400
Primary particle diameter [nm] ± SD^a,b^	10.8 ± 6.6	21.5 + 23.9/ −17.5
Zeta potential [mV] ± SD	−25.4 ± 2.3	−29.3 ± 2.0
DLS^c^, NWMD^d^ [nm] ± SD	78 ± 9	59.9 ± 5.5
DLS, PDI	0.14 ± 0.004	0.22 ± 0.003

^a^XC-values were derived after lognormal fitting of TEM-derived raw data, ^b^SD = Standard deviation, ^c^DLS = Dynamic light scattering, ^d^NWMD = Number-weighted mean diameter.

### Injection control and nanoparticle fate inside embryos

**Figure 2:**
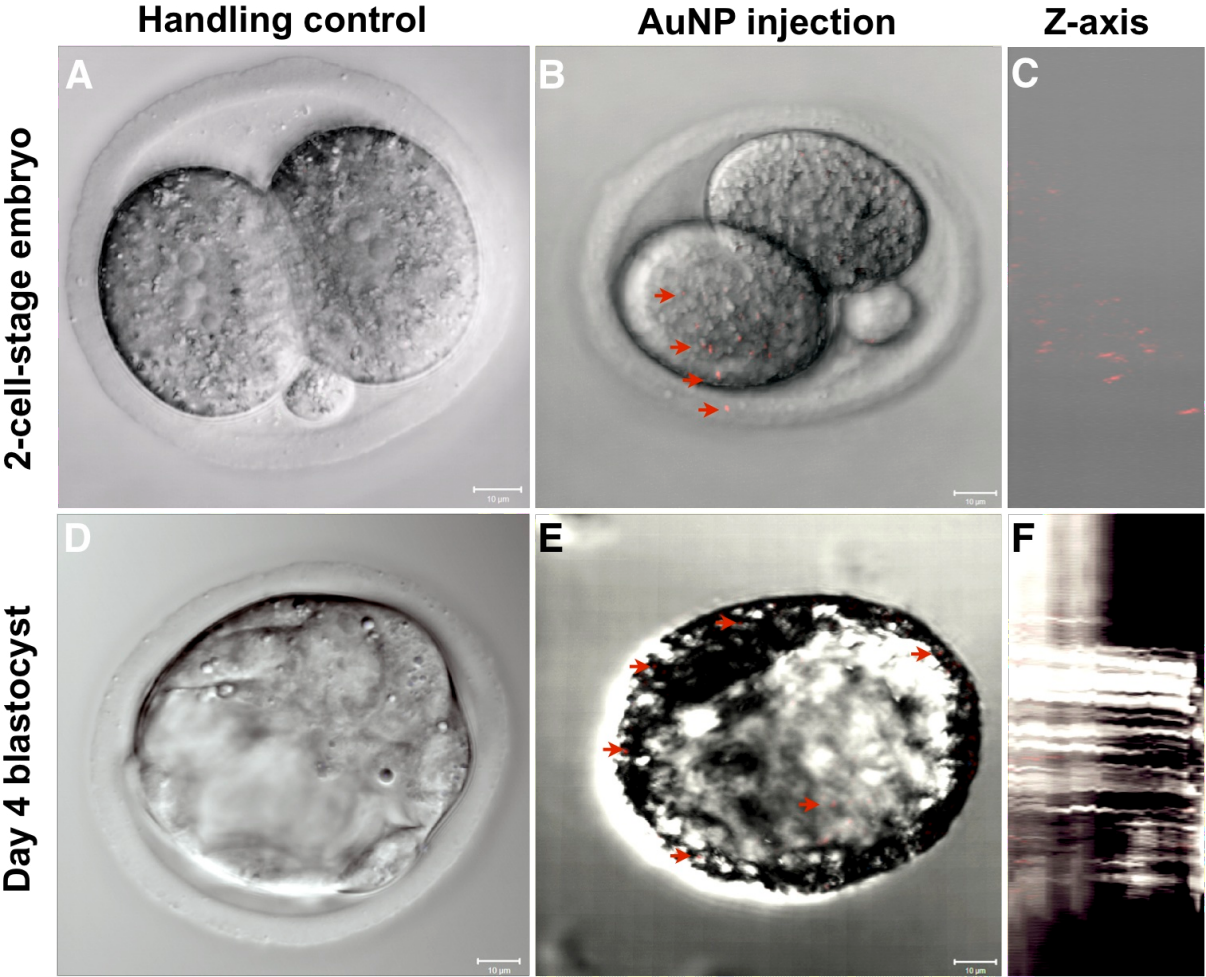
Representative laser scanning microscope images of murine embryos (projections of 10 optical sections (1 µm each)) after the injection of gold nanoparticles (10 pL of a 50 µg/mL nanoparticle dispersion, equal to 1000 nanoparticles/embryo, mean primary particle diameter as determined by TEM: 11 nm). An overlay of the differential interference contrast (DIC) merged with the gold nanoparticle detection channel is shown. Upper panels – Two-cell-stage embryos: (A) handling control, (B) embryo shortly after AuNP injection, one nanoparticle is located in the zona pellucida, highlighting the injection canal, (C) z-axis of projection (B); Lower panels – Day-four-blastocysts: (D) handling control, (E) 3 days after AuNP injection, (F) z-axis of (E). AuNP appear in red, some of which are exemplarily pointed out with arrows.

Figure 2B shows a representative image of a two-cell-stage embryo shortly after the injection of gold nanoparticles. AuNP are distributed through the entire treated blastomere, while the other remained particle-free. To appreciate the amount of injected nanoparticles, it has to be taken into account that only particles and particle agglomerates with a diameter >60 nm can be visualized by confocal microscopy [55]. The injected dispersion contained such particles only to 2.6 and 2.2% for AuNP and AgNP, respectively. Furthermore, only a medial optical section of about 10 µm is shown from the total embryos. Thus, the number of particles actually injected is considerably higher than the confocal image suggests. In some cases particles could also be observed in the perivitelline space after injection, as displayed in Figure 2B. This is due to the transjector, which operates with backpressure and always causes a minimal flow through the tip of the capillary.

Particles could be followed up all the way to the blastocyst stage (Figure 2E). However, due to an increased background the offset needed to be adjusted, which ultimately led to fewer visible particles compared to the two-cell-stage embryo.

### Embryo development

**Figure 3:**
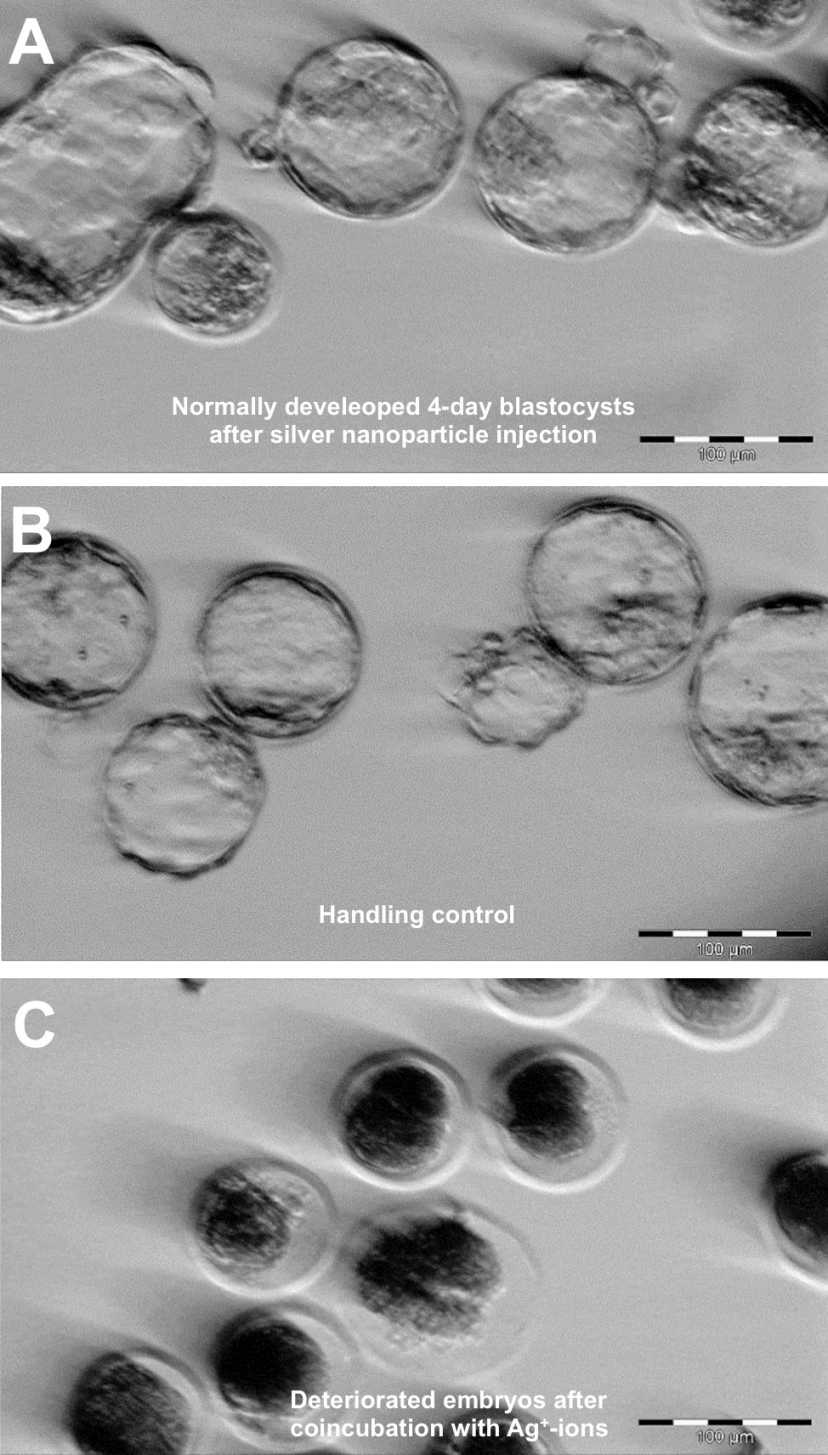
Representative stereo microscope images of murine blastocysts (A) after silver nanoparticle-injection (10 pL of a 50 µg/mL nanoparticle dispersion, equal to 3300 nanoparticles/embryo, mean primary particle diameter as determined by TEM: 21 nm), (B) untreated handling control, (C) deteriorated 2-cell-stage embryos after co-incubation with silver ions (25 µM AgNO_3_).

Neither the NP-injected nor the non-injected embryos displayed any abnormal development (Figure 3A and Figure 3B). During the daily observations of the developing embryos no indication was found that the injected blastomere showed atrophic behavior. Developmental rates are summarized in Table 2. After the injection with gold and silver nanoparticles the development rate did not differ from the sham-injected embryos or the non-injected handling controls. Control experiments with Ag^+^-ions resulted in an immediate arrest of development (Figure 3C). Silver ions were included in the dose study by adding 25 µM of AgNO_3_ to the culture medium, which is equivalent to approximately 50% of the Ag mass concentration inside the AgNP injected blastomere – given that 10 pL of a 463 µM [50 µg/mL] AgNP dispersion was added to a blastomere with a volume of 90 pL [56]. The chosen concentration of Ag^+^-ions was approximated based on previously reported ion release kinetics of silver colloids in aqueous solution [57]. Control co-incubations of embryos with equimolar KNO_3_ showed no effect.

**Table 2:** Preimplantation development rates in the various treatment groups.

Treatment	Embryos in culture (*n*)	Blastocysts (*n*)	Blastocyst rate [%]

AuNP injection	107	72	67.3^a^
AgNP injection	91	56	61.5^a^
Sham injection	74	49	66.2^a^
AgNO_3_ co-incubation	41	0	0^b^
KNO_3_ co-incubation	40	32	80^a^
Handling control	102	81	79.4^a^

^a,b^different symbols indicate significant differences (p < 0.05)

### Gene expression in developed blastocysts

**Figure 4:**
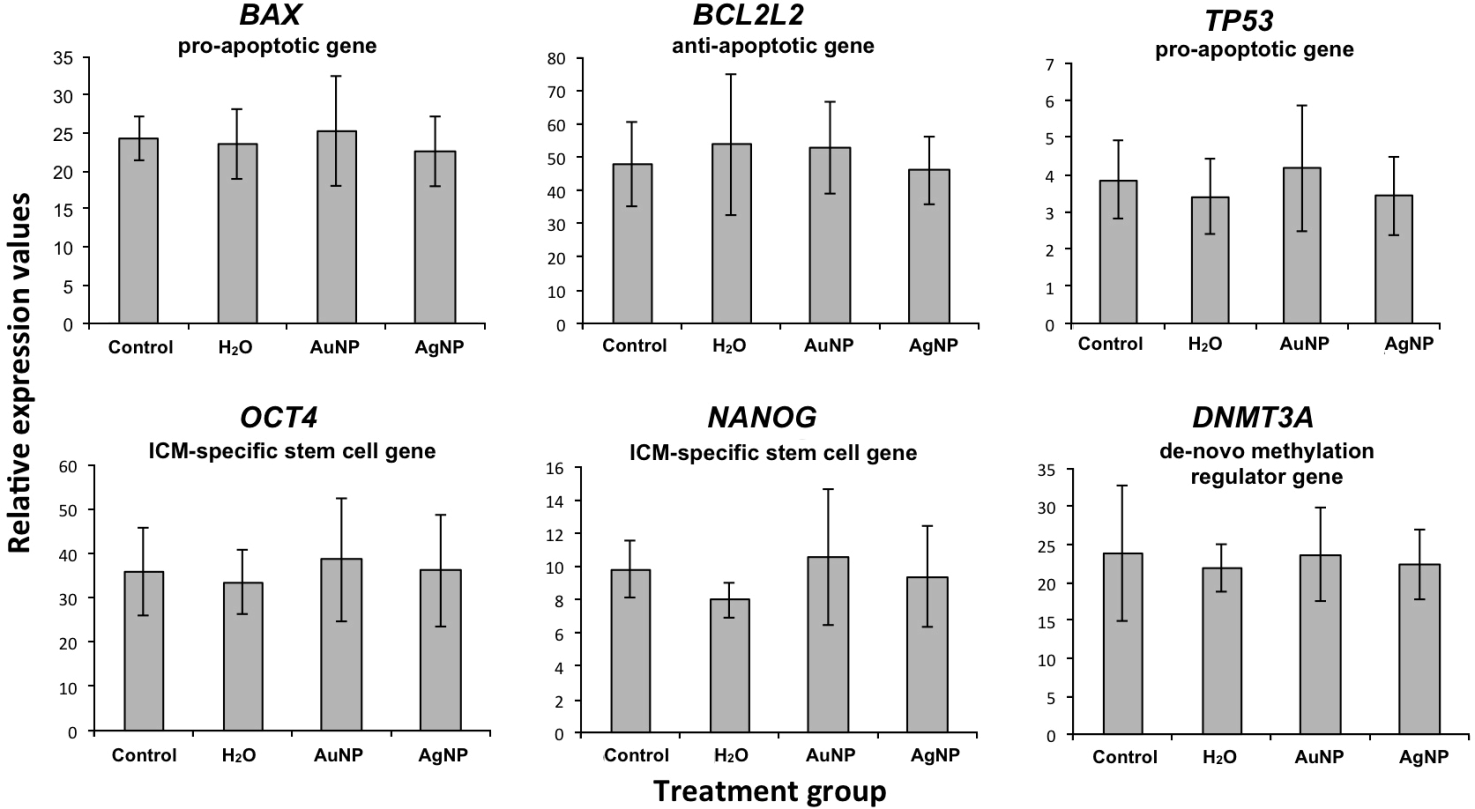
Gene expression after normalization based on globin/beta-actin transcript abundance. Values are mean ± SD.

For all injected treatment groups and the handling controls, the gene expression of candidate genes relevant for embryo development was determined. For none of the genes differential expression between treatment groups was detected. Relative expression values are displayed in Figure 4.

## Discussion

The embryonic stage is one of the most vulnerable phases for every organism. Nanoparticles, which are comparable in size to biochemical macromolecules, were proven to enter a multitude of cell types [51,58] and to cross various biological barriers [12,59–62]. Thus, a solid understanding of the toxic potential of these highly reactive particles should be obtained.

The present study shows that the direct injection of colloidal gold or silver nanoparticles into a blastomere had no effect on the development of murine embryos. Embryos developed normally after injection, without an indication for atrophy in the injected blastomere. Furthermore, particles were found inside cells belonging to the trophoblast as well as inner cell mass, pointing out that (i) the injected blastomeres were able to undergo mitoses and (ii) the daughter cells derived from the nanoparticle treated blastomeres could facilitate the first steps of differentiation, i.e., into trophoblast and inner cell mass. These findings were confirmed by the results of quantitative real-time PCR measurements. The nanoparticle-injected embryos showed no indication of changes in apoptotic activity (based on the expression of the pro-apoptotic genes *BAX* and *TP53*, the anti-apoptotic gene *BCL2L2*), of disturbances undergoing mitosis and differentiation (based on the expression of the ICM-specific genes *OCT4* and *NANOG*) or of epigenetic aberrances (based on the expression of *DNMT3A*, one of the genes responsible for the de-novo DNA methylation of the embryonic genome). Nevertheless, to further confirm these findings future experiments may additionally include the determination of blastocyst cell numbers, further genetic markers for normal development, like trophectodermal transcripts, and finally embryo transfer and full development.

The results for gold nanoparticles are in accordance with most previous studies using piscine, avian as well as murine embryos [13–14,26,38]. Reports stating reprotoxicological effects of gold are generally rare. Spermatozoa seem to be slightly more sensitive [63–64], but only one study reported embryotoxic effects of gold clusters after application of a tremendously high dose (10^14^ NP/embryo) [15]. Thus, our findings confirm the presumption that gold nanoparticles are highly biocompatible and can safely be developed for biomedical applications, such as novel biomarkers, cancer imaging and therapy as well as drug delivery [2,65].

For silver nanoparticles the results are somewhat more surprising. In piscine embryos, grave defects were found in several trials exploring the fate of embryos after co-incubation with silver nanoparticles, even with concentrations below what has been used in the current trial [13,16–20]. Considerable toxicity was also reported by the only study so far testing silver nanoparticles on murine embryos [37]. The differences between the two mouse studies might be due to the tremendously higher dose per embryo, compared to the current trial (625 ng Ag/embryo versus 0.0005 ng Ag/embryo). However, since Li et al. [37] exposed AgNP via co-incubation and the exact amount of silver actually inside the embryos was not quantified, a direct comparison of the two trials is not possible. Interestingly, studies testing the toxicity of silver nanoparticles on chicken embryos found no detrimental effects [28–30]. By injecting AgNP in a concentration of 50 µg/mL in ovo, dose and application route in these trials was comparable to the here presented experiments. Especially the similarities regarding the application route are intriguing. In all published trials where silver nanoparticle exposure to embryos was realized by co-incubation, a considerable toxicity was denoted. The co-incubations described in literature were always performed in serum-free media, thus prohibiting a protein corona to be formed around the particle. Notably, in all trials where silver nanoparticles were injected directly, no toxicity was observed. Inside embryos or chicken egg albumen, proteins are abundant, and a protein corona is probably formed immediately around the injected particles. Such protein coronas have been described and characterized for instance after exposure of nanoparticles to blood plasma and are the result of protein adsorption to the particle surface [66]. These coronas largely define the biological identity of the particle [67–68]. They have also been reported to reduce the cytotoxicity of nanomaterials [69–70]. Therefore, one hypothesis could be that in the current study a corona of intracellular proteins formed immediately around the microinjected (initially ligand-free) particles, which served to protect the embryo from detrimental nanoparticle interactions. On the other hand, the injection of silver nanoparticles into the blood stream of adult rats did result in toxic effects, even though in these trials immediate contact to proteins was given, too [71–72]. It should be considered, however, that much higher dosages were applied in these tests. This could imply that a protein corona does not completely abolish silver nanoparticle toxicity, but may raise the toxic threshold. In which way the presence of a protein corona influences embryotoxicity has not yet been explored. For example, it has been shown that such a corona has a considerable impact on the zeta potential of the particles [73]. The zeta potential has been reported to influence cytotoxicity [74–75] as well as colloidal stability [76], which in turn has also been noted to impact the toxicity of nanoparticles [40–41]. In general, it can be said, that a high positive zeta potential as well as a high colloidal stability are connected to an increase in toxicity. However, in how far these aspects changed in the nanoparticles used in the study presented here after exposure to proteins can only be speculated about, since neither zeta potential nor colloidal stability can be unbiasedly determined after an injection into the embryo.

An indication for a protective mechanism can possibly be drawn from a control experiment performed in the course of the current trial. Since the toxicity of silver nanoparticles is to a large extend attributable to silver ions dissolving from nanomaterial compounds [77], we controlled this effect by co-incubating two-cell-stage embryos with silver ion concentrations of 25 µM, which is equivalent to approximately 50% of the Ag concentration inside the AgNP injected blastomere. These embryos showed an immediate arrest of development confirming that silver ions indeed have a detrimental effect. Since no such effects were observed after AgNP injection, silver ion dissolution seems to be either significantly reduced or the ions are deactivated after injection into the embryo. This could be caused by the aforementioned proteins, which are known to deactivate heavy metal ions by complexation. Since no such protective (ion capturing) layer would have formed in the serum-free co-incubation trials, this may explain the apparent discrepancy in terms of toxicity between the different experimental set ups.

Another aspect to be taken into account is the origin of the nanoparticles used in the different trials testing the embryo-toxicity of silver nanoparticles. All trials exploring the subject in piscine embryos and the study performed on mice used particles derived by chemical means. Despite post-production purification steps, such particle dispersions contain remnants of stabilizing and reducing agents, which can unfold toxic properties of their own [78]. The studies performed on avian embryos and the experiments presented here employed particles synthesized by physical means, an electric non-explosive method and laser ablation in water, respectively. Those methods produce colloids completely free of any surfactants or ligands.

Surfactants might have a great impact on nanoparticle toxicity. For instance, only changing the strength of ligand affinity to the nanoparticle, without changing its size or charge, significantly affects the toxicity even for gold nanoparticles. This difference in soft and hard ligand corona effect has been recently reported and confirmed by molecular modelling to be caused by the blocking of ion channels by gold nanoparticles in embryonic kidney cells, with a stronger bio-response for the nanoparticles with the weaker bound ligands [39]. In organisms as sensitive as developing embryos even minute amounts of toxic material may have an impact and therefore cannot be excluded as a reason for the differences between the studies.

## Conclusion

In conclusion, the present study confirms the high biocompatibility of ligand-free gold nanoparticles even in the sensitive area of mammalian embryo pre-implantation development. These findings suggest that these versatile particles may be suitable for further development with the aim of ultimately leading to biomedical or biotechnological applications. Equally interesting are the results regarding silver nanoparticles in contrast to silver ions, which imply that their toxicity can be reduced by either adapting surface properties or choosing alternative synthesis methods, so that their use could be rendered more safely in a plethora of applications.

## Experimental

### Nanoparticle production and characterization

The applied laser-based approach to nanoparticles consists in the ablation of a target in liquid media by intense laser radiation, leading to an ejection of its constituent and the formation of a colloidal nanoparticle solution (Figure 1A), released into pure water after the collapse of the laser-induced cavitation bubble [45,79–81]. The laser and process parameters were set as previously reported [43].

The resulting AuNP and AgNP colloids were characterized directly after synthesis by UV–vis spectroscopy (Shimadzu 1650, Shimadzu Europe GmbH, Duisburg, Germany), and transmission electron microscopy (TEM; EM 10 C electron microscope, Zeiss, Oberkochen, Germany). Five hundred nanoparticles were counted and measured for the determination of the average particle diameter. The AuNP concentration was estimated by weighing (Sartorius M3P-000V001, Sartorius AG, Göttingen, Germany) the gold foil three times, before and after laser ablation with an accuracy of 1 µg.

Zeta potential measurements for the determination of the stability as well as the detection of the hydrodynamic diameter and the polydispersity index of the colloids were performed by dynamic light scattering with the Zetasizer ZS (Malvern Instruments Ltd, Worcestershire, United Kingdom). The average value of three consecutive measurements was then taken for documentation.

### Isolation of murine 2-cell embryos and microinjection

NMRI mice were kept in a conventional facility at a constant temperature of 20 °C, a constant humidity of 60%, and a 12 h light schedule (6:00–18:00). The mice were maintained and handled according to international and German animal welfare guidelines, and all experiments were approved by an external animal welfare committee at the Niedersächsisches Landesamt für Verbraucherschutz und Lebensmittelsicherheit in Oldenburg, Germany (AZ 33.9-42502-04-09/1718). For the isolation of murine 2-cell embryos, female NMRI mice were injected i.p. with pregnant mare's serum gonadotropin (PMSG, 10 U), followed by an injection of human chorionic gonadotropin (hCG, 10 U) 46–48 hours later. Then single females were mated with fertile males. The next day (day 0.5) the females were checked for a copulation plug. Plugged females were sacrificed at day 1.5, the oviduct was isolated and flushed with pre-warmed M2 medium. Two-cell embryos were collected in a drop of M2-medium (Sigma-Aldrich), and groups of 5 embryos were transferred on a glass slide placed under a microscope (Zeiss Axiovert 35M) equipped with micromanipulators. NPs at a final concentration of 50 µg/mL in distilled water were backfilled in glass injection capillaries. Individual 2-cell embryos were fixed by suction to a holding pipette, while the injection capillary was pushed through the Zona pellucida and the cell membrane. Approximately 10 pL were then injected into the cytoplasm of one blastomere by using an Eppendorf transjector 5246 (Eppendorf, Hamburg, Germany), while the other blastomere was not treated [47]. This equals with regard to the AuNP and AgNP dispensions to approximately 1000 and 3300 particles per embryo, respectively. Additionally, embryos were sham-injected with sterile filtered aqua bidest, the solvent of the nanoparticle dispersion. Untreated embryos served as handling control. The number of embryos per treatment group is detailed in Table 2. Subsequently, the embryos were cultured in M16 medium (Sigma-Aldrich) at 37 °C in a humidified incubator with 5% CO_2_ in air for 3 days. In order to distinguish whether possible effects are caused by the nanoparticles as such or by Ag^+^-ions released from the nanoparticles, additional embryos were co-incubated with 25 µM silver nitrate. To exclude the influence of the NO_3_^−^-ions on the embryo development 25 µM potassium nitrate controls were also run. Embryo development was assessed on a daily basis and documented by using a stereo-microscope (Olympus SZX16, Olympus, Hamburg, Germany) equipped with a camera (Olympus DP72, Olympus, Hamburg, Germany).

#### Laser scanning confocal microscopy (LSCM)

For nanoparticle imaging purposes, embryos were transferred onto a glass slide within a droplet of PBS without further fixation and examined immediately. Light microscopical visualization of AuNP was performed as previously described by using an Axioplan 200 and a confocal imaging system LSM510 (Carl Zeiss MicroImaging GmbH, Jena, Germany) [55]. Briefly, a helium neon green laser of 543 nm was used to excite the surface plasmon resonance of the gold nanoparticles and a 633 nm helium neon green laser for visualization of the two blastomers in differential interference contrast. Visualization of light scattering for each of the excitation wavelengths was recorded in multi-tracking mode using separate detection channels.

#### Real time-PCR

**Figure 5:**
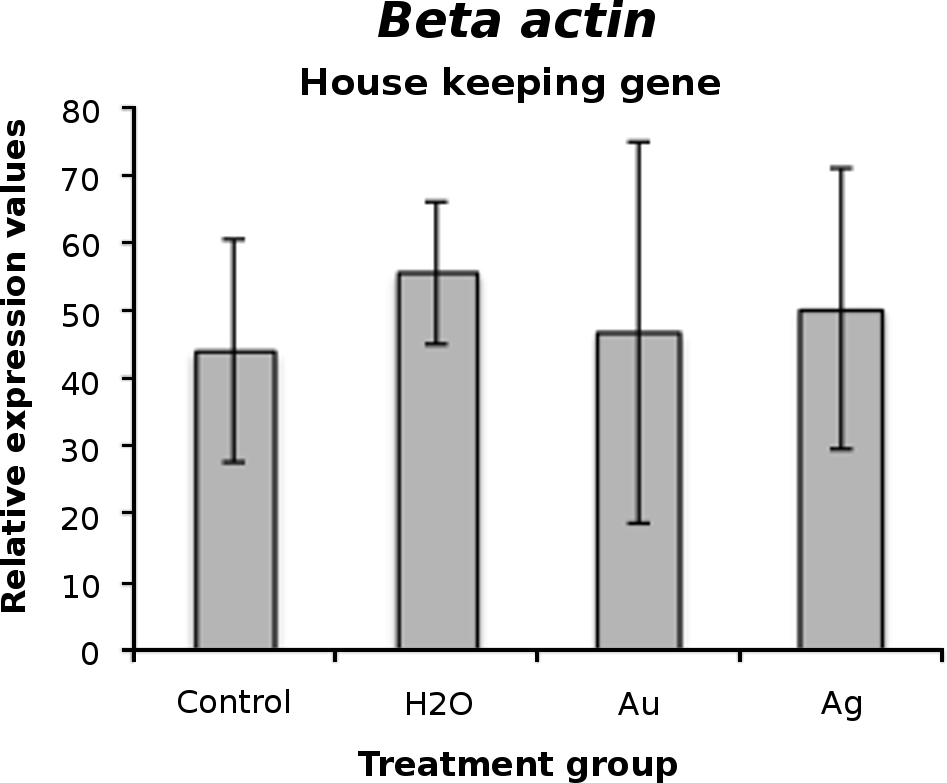
Beta actin expression after normalization with globin. Values are mean ± SD.

Real time-PCR measurements were carried out on blastocysts derived from the following treatment groups: AuNP injected, AgNP injected, sham injected, handling control. Blastocysts were pooled in groups of ten. Per treatment group, 7 pools were examined. Pools were lysed in 40 µL of lysis-binding buffer, then 1 pg of rabbit globin mRNA (BRL, Gaithersburg, MD) was added as an external standard. Poly(A)+-RNA was prepared with a Dynabeads^®^ mRNA Direct Kit (Dynal, Oslo, Norway). Reverse transcription (RT) was performed in a 20 µL volume consisting of 2 µL of 10× RT buffer (Invitrogen), 2 µL of 50 mM MgCl_2_ (Invitrogen), 2 µL of 10 mM dNTP solution (Amersham Biosciences), 1 µL (20 U) of RNAsin (Applied Biosystems), 1 µL (50 U) of RT from Moloney Murine Leukemia Virus (Applied Biosystems), 1 µL random hexamers (50 µM) (Applied Biosystems), and 11 µL of millipore purified destilled water. The samples were incubated at 25 °C for 10 min, then at 42 °C for 1 h, and finally at 95 °C for 5 min. The PCR mix in each well included 10 µL of 2× Power SYBR_Green PCR Master Mix (Applied Biosystems), 6.4 µL dH_2_O, 1.6 µL each of the primer pairs (5 µM), 2 µL of cDNA in a volume of 20 µL. Primer and PCR characteristics are summarized in Table 3. The PCR program included denaturation and activation of the Taq polymerase for 10 min at 95 °C followed by 43 cycles of 95 °C for 15 s and the appropriate annealing temperature given in Table 3 for 1 min and finally heating with a ramp rate of 2%: 95 °C for 15 s, 60 °C for 15 s and 95 °C for 15 s to display a dissociation curve of the product (ABI 7500 Fast Real-Time System, Applied Biosystems). Results are given as relative expression values derived from standard curve calculated quantities after globin based correction and normalization on beta actin expression. Beta actin expression was steady as displayed in Figure 5.

**Table 3:** Realtime PCR primers and characteristics.

Symbol	Sequence (5’-3’, forward and reverse)	Amplicon size	Annealing *t*	RTPrimerDB ID [70]

*ACTb*	CAACGAGCGGTTCCGATG (18 bp) GCCACAGGATTCCATACCCA (20 bp)	67 bp	60 °C	2847
*Globin*	GCAGCCACGGTGGCGAGTAT (20 bp) GTGGGACAGGAGCTTGAAAT (20 bp)	256 bp	60 °C	Heinzmann et al. [71]
*BCL2l2*	GTTTCCGCCGCACCTTCTCT (20 bp) CCCCGTCAGCACTGTCCTCA (20 bp)	362 bp	59 °C	Exley et al. [72]
*BAX*	ATGCGTCCACCAAGAAGCTGA (21 bp) AGCAATCATCCTCTGCAGCTCC (22 bp)	86 bp	60 °C	2868
*TRP53*	TGAAACGCCGACCTATCCTTA (21 bp) GGCACAAACACGAACCTCAAA (21 bp)	92 bp	60 °C	3365
*DNMT3A*	CGGCAGAATAGCCAAGTTCA (20 bp) CTGGTCTTTGCCCTGCTTTA (20 bp)	76 bp	60 °C	8144
*OCT4*	GAAGCAGAAGAGGATCACCTTG (22 bp) TTCTTAAGGCTGAGCTGCAAG (21 bp)	129 bp	58 °C	3577
*NANOG*	CCTCAGCCTCCAGCAGATGC (20 bp) CCGCTTGCACTTCACCCTTTG (21 bp)	100 bp	58 °C	3576

#### Statistical analysis

Statistical analysis was performed with JMP version 7.0 (SAS Institute, Inc., Cary, NC) and R, version 2.15.2 [82]. To study the effect of gold and silver nanoparticles on gene expression a one way analysis of variance was applied. The influence of treatment on blastocyst development rates was investigated by chi square test and pairwise comparisons of proportions taking corrections for multiple testing into account.
